# Monocyte count as an independent predictor of high thrombus burden in ST-elevation myocardial infarction patients undergoing percutaneous coronary intervention: A systematic review and meta-analysis

**DOI:** 10.1097/MD.0000000000041132

**Published:** 2025-01-17

**Authors:** Hassan ul Hussain, Kanwal Ashok Kumar, Marium Zahid, Muhammad Husban Burney, Muqaddus Asif, Syeda Tayyaba Rehan, Khabab Abbasher Hussien Mohamed Ahmed, Irfan Ullah

**Affiliations:** a Dow University of Health Sciences, Karachi, Pakistan; b Karachi Medical and Dental College, Karachi, Pakistan; c Faculty of Medicine, University of Khartoum, Khartoum, Sudan; d Kabir Medical College, Gandhara University, Peshawar, Pakistan; e Institute of Public Health & Social Sciences (IPH&SS), Khyber Medical University, Peshawar, Pakistan.

**Keywords:** monocyte count, percutaneous coronary intervention, STEMI, thrombosis

## Abstract

**Background:**

Due to the lack of a prior comprehensive review and meta-analysis, the relationship between monocyte count and thrombus load in ST-elevation myocardial infarction (STEMI) patients undergoing percutaneous coronary intervention (PCI) has not been adequately established.

**Methods:**

This was a systematic review and meta-analysis of multiple cohorts (retrospective and prospective) and cross-sectional studies.

We queried electronic databases (PubMed, Google Scholar, and Cochrane Central) from their inception to April 2022. The included studies had patients who had undergone PCI treatment and were classified using thrombolysis in myocardial infarction thrombus grading. Dichotomous outcomes from the studies were presented as odds ratios with 95% confidence intervals while means ± standard deviation were presented for continuous outcomes. Means ± standard deviation of monocyte levels and odds ratios were pooled using an inverse variance-weighted random-effects model. *I*² statistics are used to evaluate heterogeneity across studies and subgrouping was performed to reduce the heterogeneity.

**Results:**

Five eligible studies, consisting of 1426 patients were included, out of which 776 had a high thrombus burden. Pooled results after subgroup analysis showed a significant relationship between raised monocyte count and high thrombus burden (odds ratios = 1.44; 95% confidence interval = 1.06–1.96; *P* = .02; I2 = 71%). Post-subgroup pooled analysis revealed a statistically significant correlation between high thrombus burden and raised monocyte count.

**Conclusion:**

STEMI patients with a high thrombus burden show an increased monocyte count. A high monocyte count upon admission is a major indication of increased intracoronary thrombus burden in STEMI patients with PCI procedures.

## 1. Introduction

Heart disease, especially coronary artery disease, has been identified as one of the leading causes of death worldwide in recent years.^[1]^ In patients with coronary artery disease, ST-elevation myocardial infarction (STEMI) is a substantial source of morbidity and mortality.

Myocardial damage or necrosis comes from transmural ischemia, which is caused by STEMI of 1 or more coronary arteries.^[2]^ An intracoronary thrombus that develops as a result of an atherosclerotic plaque rupture is one of the leading causes of STEMI. Percutaneous coronary intervention (PCI) is one of the primary therapeutic modalities for STEMI patients.^[3]^

In addressing STEMI, evaluation of intracoronary thrombus load may be helpful. A thrombus scoring method for thrombolysis in myocardial infarction (TIMI) can be used to determine thrombus burden.^[4]^ A combination of a thrombus grade of >2 on the TIMI scale, evidence of a distal embolism on arteriography, and/or the presence of sluggish flow without embolism or dissection is referred to as an intracoronary thrombus load.^[5]^ A higher TIMI score predicts a greater risk of fatalities or serious cardiac events.^[6]^

According to Wang et al retrospective cohort study, monocytes have a role in the pathophysiology of STEMI by causing thrombotic problems. They accomplish this by secreting inflammatory processes and procoagulant substances such as tissue factors. A myocardial infarction risk factor is an increased monocyte count.^[7]^ Numerous cytokines that regulate inflammatory processes are produced by monocytes. According to research, monocytes can control the production of inflammatory cytokines and the remodeling of tissue during the creation of thrombi.^[8]^

Monocyte count and thrombus burden have been linked in a few isolated studies conducted primarily in the Middle East and Asia up to this point, but there has not been a comprehensive analysis of these studies conducted anywhere else in the world, making it difficult for clinicians to apply the findings to the care of STEMI patients.

In STEMI patients undergoing PCI, the main goal of this systematic review and meta-analysis is to ascertain the relationship between monocyte count and thrombus burden. Evidence gathered in this analysis has the potential to revolutionize emergency medicine and pave the way for a multitude of more studies in this area.

## 2. Materials and methods

This study was conducted by the reporting guidelines set by preferred reporting items for systematic review and meta-analysis (PRISMA).^[9]^ PRISMA checklist has been provided in Table 1. As this is a compilation of publicly accessible results, no institutional review board permission or patient-informed consent was required. This systematic review and meta-analysis have been registered on Prospero. ID: CRD42022328540.

**Table 1:** PRISMA checklist (from page MJ, McKenzie JE, Bossuyt PM, Boutron I, Hoffmann TC, Mulrow CD, et al. The PRISMA 2020 statement: an updated guideline for reporting systematic reviews. BMJ 2021;372:n71. doi: 10.1136/bmj.n71. For more information, visit: http://www.prisma-statement.org/).

Section and topic	Item #	Checklist item	Location where item is reported
Title			
Title	1	Identify the report as a systematic review.	1
Abstract			
Abstract	2	See the PRISMA 2020 for Abstracts checklist.	2
Introduction			
Rationale	3	Describe the rationale for the review in the context of existing knowledge.	3, 4
Objectives	4	Provide an explicit statement of the objective(s) or question(s) the review addresses.	3, 4
Methods			
Eligibility criteria	5	Specify the inclusion and exclusion criteria for the review and how studies were grouped for the syntheses.	4, 5
Information sources	6	Specify all databases, registers, websites, organizations, reference lists and other sources searched or consulted to identify studies. Specify the date when each source was last searched or consulted.	4, 5
Search strategy	7	Present the full search strategies for all databases, registers and websites, including any filters and limits used.	4, 5
Selection process	8	Specify the methods used to decide whether a study met the inclusion criteria of the review, including how many reviewers screened each record and each report retrieved, whether they worked independently, and if applicable, details of automation tools used in the process.	4, 5
Data collection process	9	Specify the methods used to collect data from reports, including how many reviewers collected data from each report, whether they worked independently, any processes for obtaining or confirming data from study investigators, and if applicable, details of automation tools used in the process.	4, 5
Data items	10a	List and define all outcomes for which data were sought. Specify whether all results that were compatible with each outcome domain in each study were sought (e.g., for all measures, time points, analyses), and if not, the methods used to decide which results to collect.	4, 5
	10b	List and define all other variables for which data were sought (e.g., participant and intervention characteristics, funding sources). Describe any assumptions made about any missing or unclear information.	4, 5
Study risk of bias assessment	11	Specify the methods used to assess risk of bias in the included studies, including details of the tool(s) used, how many reviewers assessed each study and whether they worked independently, and if applicable, details of automation tools used in the process.	4, 5
Effect measures	12	Specify for each outcome the effect measure(s) (e.g., risk ratio, mean difference) used in the synthesis or presentation of results.	4, 5
Synthesis methods	13a	Describe the processes used to decide which studies were eligible for each synthesis (e.g., tabulating the study intervention characteristics and comparing against the planned groups for each synthesis (item #5)).	4, 5
	13b	Describe any methods required to prepare the data for presentation or synthesis, such as handling of missing summary statistics, or data conversions.	4, 5
	13c	Describe any methods used to tabulate or visually display results of individual studies and syntheses.	4, 5
	13d	Describe any methods used to synthesize results and provide a rationale for the choice(s). If meta-analysis was performed, describe the model(s), method(s) to identify the presence and extent of statistical heterogeneity, and software package(s) used.	4, 5
	13e	Describe any methods used to explore possible causes of heterogeneity among study results (e.g., subgroup analysis, meta-regression).	4, 5
	13f	Describe any sensitivity analyses conducted to assess robustness of the synthesized results.	4, 5
Reporting bias assessment	14	Describe any methods used to assess risk of bias due to missing results in a synthesis (arising from reporting biases).	4, 5
Certainty assessment	15	Describe any methods used to assess certainty (or confidence) in the body of evidence for an outcome.	4, 5
Results			
Study selection	16a	Describe the results of the search and selection process, from the number of records identified in the search to the number of studies included in the review, ideally using a flow diagram.	5–10
	16b	Cite studies that might appear to meet the inclusion criteria, but which were excluded, and explain why they were excluded.	5–10
Study characteristics	17	Cite each included study and present its characteristics.	5–10
Risk of bias in studies	18	Present assessments of risk of bias for each included study.	5–10
Results of individual studies	19	For all outcomes, present, for each study: (a) summary statistics for each group (where appropriate) and (b) an effect estimate and its precision (e.g., confidence/credible interval), ideally using structured tables or plots.	5–10
Results of syntheses	20a	For each synthesis, briefly summarize the characteristics and risk of bias among contributing studies.	5–10
	20b	Present results of all statistical syntheses conducted. If meta-analysis was done, present for each the summary estimate and its precision (e.g., confidence/credible interval) and measures of statistical heterogeneity. If comparing groups, describe the direction of the effect.	5–10
	20c	Present results of all investigations of possible causes of heterogeneity among study results.	5–10
	20d	Present results of all sensitivity analyses conducted to assess the robustness of the synthesized results.	5–10
Reporting biases	21	Present assessments of risk of bias due to missing results (arising from reporting biases) for each synthesis assessed.	5–10
Certainty of evidence	22	Present assessments of certainty (or confidence) in the body of evidence for each outcome assessed.	5–10
Discussion			
Discussion	23a	Provide a general interpretation of the results in the context of other evidence.	10, 11
	23b	Discuss any limitations of the evidence included in the review.	10, 11
	23c	Discuss any limitations of the review processes used.	10, 11
	23d	Discuss implications of the results for practice, policy, and future research.	10, 11
Other information			
Registration and protocol	24a	Provide registration information for the review, including register name and registration number, or state that the review was not registered.	12
	24b	Indicate where the review protocol can be accessed, or state that a protocol was not prepared.	12
	24c	Describe and explain any amendments to information provided at registration or in the protocol.	12
Support	25	Describe sources of financial or nonfinancial support for the review, and the role of the funders or sponsors in the review.	12
Competing interests	26	Declare any competing interests of review authors.	12
Availability of data, code and other materials	27	Report which of the following are publicly available and where they can be found: template data collection forms; data extracted from included studies; data used for all analyses; analytic code; any other materials used in the review.	12

PRISMA = preferred reporting items for systematic review and meta-analysis.

### 2.1. Data sources and search strategy

Three Electronic databases named PubMed, Google Scholar, and Cochrane Central were searched from their inception till April 2022, without placing any language and time restrictions. We used the medical subject headings “Monocytes,’’ “Thrombus,’’ “Blood clot,” “STEMI,” ST-segment elevation myocardial infarction,” ST elevated myocardial infarction.” A detailed search string for each database has been provided in Table 2. We searched for gray-and-white literature. Bibliographies of retrieved trials, meta-analyses, and systematic reviews were also hand-searched to ensure no relevant articles were overlooked.

**Table 2:** Search strategy used in each database.

Database	Search strategy	Obtained articles
PubMed	(“Monocytes”[All Fields] OR “Monocyte”[All Fields] OR “Monocytic”[All Fields]) AND (“Thrombosis”[All Fields] OR “Thrombus”[All Fields] OR “Thrombotic”[All Fields] OR “Blood clot”[All Fields]) AND (“STEMI”[All Fields] OR “ST-Segment Elevation Myocardial Infarction”[All Fields] OR “ST Elevated Myocardial Infarction”[All Fields])	31
Cochrane Central	(“Monocytes” OR “Monocyte” OR “Monocytic” AND “Thrombosis” OR “Thrombus” OR “Thrombotic” OR “Blood clot” AND “STEMI” OR “ST-Segment Elevation Myocardial Infarction” OR “ST Elevated Myocardial Infarction”)	14,976
Google Scholar	(“Monocytes” OR “Monocyte” OR “Monocytic” AND “Thrombosis” OR “Thrombus” OR “Thrombotic” OR “Blood clot” AND “STEMI” OR “ST-Segment Elevation Myocardial Infarction” OR “ST Elevated Myocardial Infarction”)	9690

### 2.2. Study selection

Following were the prespecified eligibility criteria for including studies: (a) Target population included STEMI patients; (b) All patients who underwent PCI; (c) Patients were grouped based on TIMI thrombus grading; (d) Studies reporting the relation of high thrombus burden with monocyte count as an outcome; (e) For comparisons of increased and decreased monocyte levels, an effect estimate (odds ratios (OR)) with 95 percent confidence intervals (CI) and/or mean ± standard deviation for monocyte level was provided; (f) Comparison group included patients with low thrombus burden.

The selected studies were exported to EndNote Reference Library Software (X7 v17.0.0.7072), where duplicates were screened and removed. The remaining articles were then blindly reviewed by 2 independent reviewers, first based on the title and abstract, and then the full text was reviewed to evaluate relevance. Studies that met the prespecified eligibility criteria were included only. All studies having irrelevant outcomes, irrelevant populations, case reports, registries, letters, or not released as published reports were excluded.

### 2.3. Data extraction and quality assessment

Study characteristics, baseline demographics, and outcome data OR for developing high thrombus burden) were extracted into a Microsoft Excel Sheet. Quality assessment for the included observational studies was done by using the Newcastle–Ottawa scale, which was based on the selection, comparability, and outcome/exposure criterion of included studies. Data extraction and quality assessment were conducted independently by 2 independent reviewers and conflicts were resolved by group discussion. The concordance rate between the reviewers was 97.5%.

### 2.4. Statistical analysis

RevMan (version 5.4; Copenhagen: The Nordic Cochrane Centre, The Cochrane Collaboration, 2014) was used to perform all statistical analyses. The results of the report expressed as means ± standard deviation were pooled using the random-effects model. The results expressed as OR with 95% CIs were pooled using the inverse variance-weighted random-effects model. Forest plots were created to visually assess the pooled results. *I*² statistics are used to evaluate heterogeneity across the studies, with a value of 25% to 50% *I*² being mild, 50% to 75% being moderate and >75% being considered severe heterogeneity.^[10]^ The outcome was severalized into subgroups based on the number of participants, male percentage, and study design to reduce the heterogeneity. A *P*-value of <.05 was considered significant in all cases. Funnel plots were not made, and Begg and Egger regression tests were not performed to assess publication bias as our meta-analysis does not contain enough studies needed to perform these tests.

## 3. Results

### 3.1. Literature search

**Figure 1.**
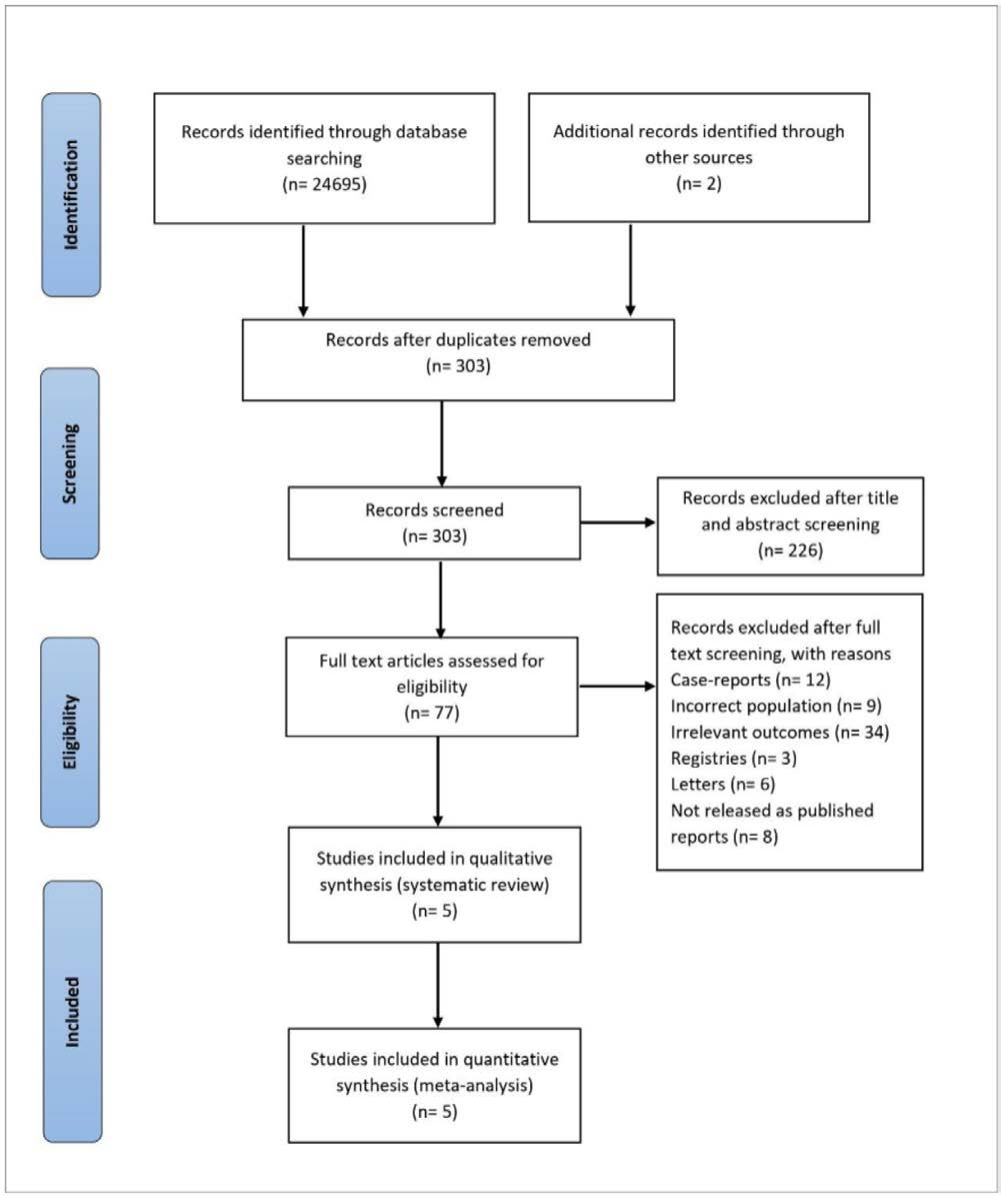
PRISMA flowchart summarizing results of literature search. PRISMA = preferred reporting items for systematic review and meta-analysis.

An initial search of 3 electronic databases (PubMed, Google Scholar, and Cochrane Central) and some other sources resulted in 24,697 articles, out of which 303 articles were left after the removal of duplicates. Two hundred twenty-six articles were then excluded based on the abstract and title screening. After a full-text review of 77 articles, 72 studies were further excluded that did not meet the inclusion criteria. Finally, 5 observational studies were recruited to be analyzed in this meta-analysis. The summary of the literature search is presented in the PRISMA flow chart (Fig. 1).

### 3.2. Study characteristics and patients’ baseline characteristics

Out of the 5 observational studies, there were 2 cross-sectional studies^[11,12]^ and 3 cohort studies.^[13–15]^ The earliest study was published in August 2016 whereas the most recent study was published in April 2022. The sample size of participants in the studies included ranged from 161 to 414. These 5 studies showed a total of 1426 participants, amongst which 776 participants reported a high thrombus burden whilst the remaining had a low thrombus burden. The thrombus burden was assessed based on the TIMI thrombus grading. According to this grading, low thrombus burden was subclassified into grade 0 (no thrombus), grade 1 (thrombus possibility), and grade 2 (definite thrombus with the greatest dimension <1/2 the vessel diameter). In contrast, high thrombus burden was subclassified into grade 3 (definite thrombus with thrombus’ greatest dimension >1/2 to <2 vessel diameters), grade 4 (definite thrombus with thrombus’ greatest dimension >2 vessel diameters), and grade 5 (total thrombotic occlusion).

Ethylenediamine tetraacetic acid tubes were used to assess complete blood count in all the studies. Automated blood cell counters (Beckman Coulter analyzer or XS-1000i) were used in 4 of the studies.^[11,13–15]^ The mean monocyte count values ranged from 0.61 ± 0.29 × 10^9^/L to 0.95 ± 0.66 × 10^9^/L. For monocyte cutoff values, 2 studies^[11,14]^ used 0.60 × 10^9^/L and 0.48 × 10^9^/L, and the other 2 studies used monocyte to HDL ratio (MHR) cutoff values of 19.7 and 2.41,^[13,15]^ respectively, whereas 1 study^[12]^ did not mention any cutoff. The proportion of males varied from 53.40% to 81.20%, with a mean of 69.16%. This meta-analysis included all significant research, and data were gathered from various regions, with 60% of the studies being from the Middle East, 20% from South Asia, and 20% from East Asia. Study characteristics and participants’ selection criteria are tabulated in Table 3 and baseline characteristics of patients are tabulated in Table 4.

**Table 3:** Characteristics of included studies.

First author (year)	Region	Study design	Participants (n)	Assessment of TB	Assessing CBC; blood analyser	Monocyte cutoff value (×10^9^/L)	MHR cutoff value	Monocyte – OR (95% CI)	Study significant?
Arisoy A (2016)^[13]^	Turkey (Middle East)	Cohort	414	TIMI scale	EDTA-anticoagulated Monovettew tubes (Sarstedt Monovette, Nuembrecht, Germany); Automated blood cell counter (Beckman Coulter analyzer; Beckman Coulter, California)	NR	19.70	0.354 (0.124–1.013)	Yes
Wang Z (2018)^[14]^	China (East Asia)	Retrospective Cohort	273	TIMI scale	EDTA; Automated blood cell counter (XS-1000i; Sysmex Co.)	0.48	NR	3.107 (1.199–7.052)	Yes
Sercelik A (2018)^[15]^	Turkey (Middle East)	Prospective Cohort	161	TIMI scale	Automated blood cell counter (Beckman Coulter analyzer, Brea, CA)	NR	2.41	NR	Yes
Separham A (2020)^[11]^	Iran (Middle East)	Cross-Sectional	400	TIMI scale	EDTA-tubes; Auto-analyzer	0.60	NR	3.099 (1.205–6.988)	Yes
Zeeshan M (2022)^[12]^	Pakistan (South Asia)	Cross-Sectional	178	TIMI scale	EDTA	NR	NR	1.318 (1.140–1.524)	Yes

CBC = complete blood count, CIs = confidence intervals, EDTA = ethylenediamine tetraacetic acid, ORs = odds ratios, MHR = monocyte to HDL ratio, TIMI scale = thrombolysis in myocardial infarction scale.

**Table 4:** Baseline characteristics of patients in included studies.

First author (year)	No. of participants (n)	LTB (n)	HTB (n)	Mean age (years ± SD)		Males (n%)		Hemoglobin (g/dL) (mean ± SD)		Hematocrit (%) (mean ± SD)		WBC (×10^9^/L) (mean ± SD)		Neutrophil count (×10^9^/L) (mean ± SD)			Lymphocyte count (×10^9^/L) (mean ± SD)		Monocyte count (×10^9^/L) (mean ± SD)		Creatinine (mg/dL)		Platelet count (×10^9^/L) (mean ± SD)		Mean platelet volume (fL)		Diabetes mellitus (n, %)		Hypertension (n, %)		Hyperlipidemia (n, %)		Active smokers (n, %)		Prior MI (n, %)	
				Low thrombus	High thrombus	Low thrombus	High thrombus	Low thrombus	High thrombus	Low thrombus	High thrombus	Low thrombus	High thrombus	Low thrombus	High thrombus	Low thrombus		High thrombus	Low thrombus	High thrombus	Low thrombus	High thrombus	Low thrombus	High thrombus	Low thrombus	High thrombus	Low thrombus	High thrombus	Low thrombus	High thrombus	Low thrombus	High thrombus	Low thrombus	High thrombus	Low thrombus	High thrombus
Arisoy A (2016)	414	181	233	62.20 ± 13.20	63.0 ± 12.50	131 (72)	184 (79)	14.60 ± 1.50	14.40 ± 1.20	NR	NR	10.80 ± 3.0	11.90 ± 3.72	7.40 ± 2.50	8.0 ± 3.20	1.83 ± 1.12		1.91 ± 1.20	0.65 ± 0.40	0.95 ± 0.66	0.91 ± 0.18	0.92 ± 0.18	226.70 ± 50.80	237.50 ± 64.20	8.80 ± 2.20	9.20 ± 1.80	38.01 (21)	55.60 (24)	56.11 (31)	65.16 (36)	70.60 (39)	97.86 (42)	77.83 (43)	95.53 (41)	45.25 (25)	32.58 (18)
Wang Z (2018)	273	178	95	62.30 ± 13.20	62.0 ± 14.70	143 (80.33)	77 (81.05)	14.40 ± 1.90	14.40 ± 2.30	42.30 ± 4.70	42.20 ± 4.90	9.60 ± 3.0	9.90 ± 3.20	6.80 ± 2.80	6.90 ± 3.30	2.23 ± 1.94		2.32 ± 1.35	0.53 ± 0.24	0.61 ± 0.29	0.86 ± 0.27	0.92 ± 0.28	214.30 ± 60.50	218.80 ± 53.80	10.30 ± 0.80	10.20 ± 0.90	56 (31.50)	31 (32.60)	106 (59.60)	57 (60.0)	109 (61.20)	65 (68.40)	75 (42.10)	47 (49.50)	6 (3.40)	3 (3.20)
Sercelik (2018)	161	39	72	50.80 ± 5.30	52.10 ± 6.90	31 (79.50)	55 (76.38)	14.80 ± 1.85	14.80 ± 1.72	NR	NR	8.9 ± 1.82	10.50 ± 2.90	5.70 ± 1.70	6.99 ± 2.93	2.20 ± 0.74		2.35 ± 0.95	0.64 ± 0.17	0.75 ± 0.26	0.86 ± 0.22	0.81 ± 0.19	245.30 ± 73.40	259.40 ± 68.40	NR	NR	7.80 (20.50)	21.02 (29.20)	9.01 (23.10)	31.03 (43.10)	NR	NR	18.01 (46.2)	32 (44.40)	7 (17.94)	18 (25)
Separham A (2020)	400	144	256	59.85 ± 11.68	58.71 ± 12.31	112 (77.77)	213 (83.20)	14.50 ± 1.90	14.60 ± 2.10	42.10 ± 4.50	42.0 ± 4.70	10.30 ± 4.0	10.40 ± 4.50	7.10 ± 1.30	6.80 ± 1.30	2.17 ± 1.08		2.23 ± 1.11	0.59 ± 0.28	0.81 ± 0.33	NR	NR	239.50 ± 67.90	234.60 ± 76.30	10.20 ± 0.60	10.10 ± 0.70	48 (33.33)	65 (25.40)	86 (59.70)	159 (62.10)	18 (12.50)	46 (18.0)	83 (57.60)	148 (57.80)	5 (3.50)	8 (3.10)
Zeeshan M (2022)	178	58	120	37.75 ± 6.39	56.04 ± 7.98	0 (0)	97 (80.83)	NR	NR	NR	NR	NR	NR	NR	NR	NR		NR	0.62 ± 0.06	0.70 ± 0.03	NR	NR	NR	NR	NR	NR	0 (0)	118 (98.33)	5 (8.63)	120 (100)	NR	NR	12 (20.69%)	120 (100%)	NR	NR

### 3.3. Quality assessment and publication bias

Quality assessment of included studies was carried out on the Newcastle–Ottawa scale, with a range of 6 to 8 out of a maximum of 9 as depicted in the quality assessment in Tables 5 and 6. Funnel plots were not made and Egger and Begg regression tests were not performed to check the relevance of small studies and publication bias as our meta-analysis was not eligible for them due to <10 studies.

**Table 5:** A detailed Newcastle–Ottawa scale of each included cohort study.

Study	Selection				Comparability		Outcome			Total quality score
	Representativeness of exposed cohort	Selection of non-exposed cohort	Ascertainment of exposure	Demonstration that outcome of interest was present or not at the start	Adjust for the most important risk factors	Adjust for other risk factors	Assessment of outcome	Follow-up length	Loss of follow-up length	
Arisoy (2016)	1	1	1	1	1	1	1	0	0	7
Wang Z (2016)	1	1	1	1	1	0	1	0	0	6
Sercelik (2018)	1	1	1	1	1	1	1	0	1	8

**Table 6:** A detailed Newcastle–Ottawa scale of each included a cross-sectional study.

Study	Selection				Comparability	Outcome		Total quality score
	Representativeness of the sample	Sample size	Nonrespondents	Ascertainment of the exposure (risk factor)	Confounding factors controlled	Assessment of outcome	Statistical test	
Separham A (2020)	1	1	1	1	2	1	1	8
Zeeshan A (2022)	1	1	1	1	2	1	1	8

### 3.4. Outcome analysis

**Figure 2.**
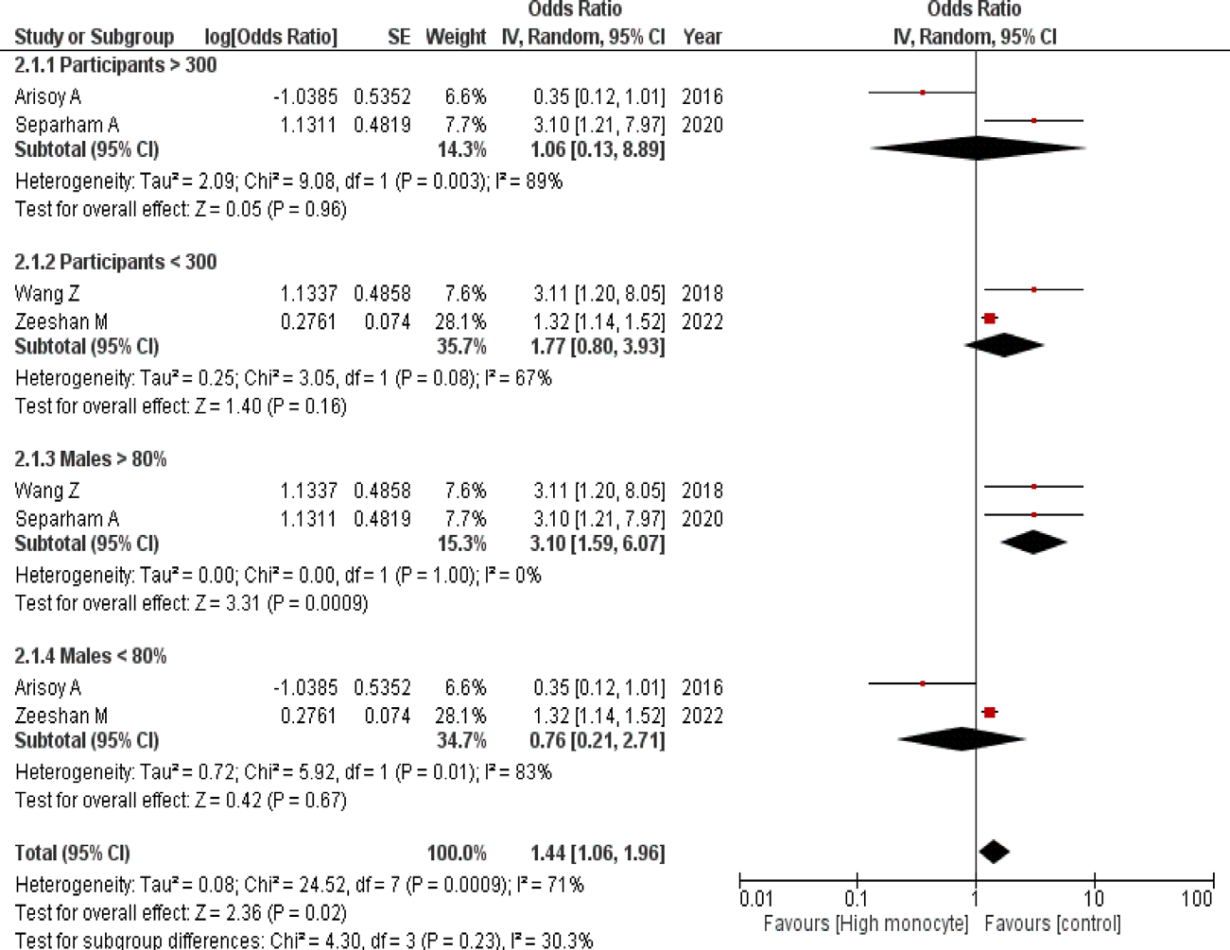
Forest plot depicting the occurrence of high thrombus burden associated with high monocyte levels. CI = confidence interval.

All 5 studies reported data on monocyte count in STEMI patients with high and low thrombus burdens. Out of these, a pooled analysis of 4 studies^[11–14]^ (1265 participants, 704 patients with high thrombus count, and 561 patients with low thrombus burden) showed an insignificant association between raised monocyte count and high thrombus burden (OR = 1.47; 95% CI = 0.70–3.08; *P* = .30, *I*^2^ = 76%) before we performed subgroup analysis. After performing a subgroup analysis based on the number of participants and gender percentages, the pooled analysis showed a significant relationship between raised monocyte count and high thrombus burden (OR = 1.44; 95% CI = 1.06–1.96; *P* = .02). Studies classified as having males >80% (OR = 3.10; 95% CI = 1.59–6.07; *P* = .0009) showed increased monocyte count amongst the patients. No significant association was noted in studies having participants >300 (OR = 1.06; 95% CI = 0.13–8.89; *P* = .96), participants <300 (OR = 1.77; 95% CI = 0.8–3.93, *P* = .16), males <80% (OR = 0.76; 95% CI = 0.21–2.71; *P* = .67). The subgrouping analysis did not reveal any significant differences in the heterogeneity (*I*^2^ = 71%, *P* = .0009) (Fig. 2).

**Figure 3.**
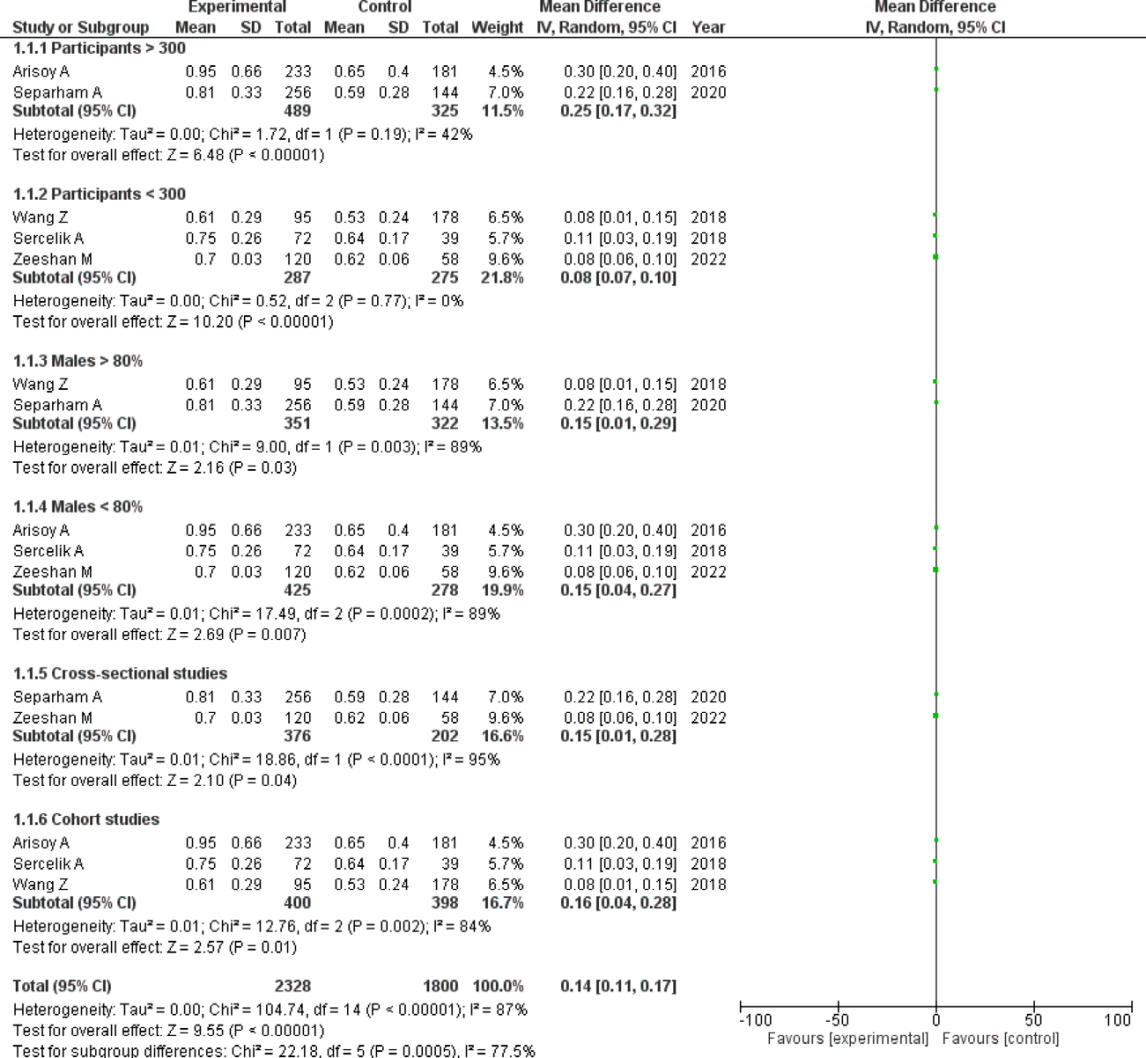
Forest plot comparing monocyte levels for high versus low thrombus burden. CI = confidence interval.

Included studies^[11–15]^ reported 1426 participants in total, 776 patients were with high thrombus burden while 600 patients were registered with low thrombus burden. This plot (Fig. 3) demonstrated a significant association of raised monocyte count among high thrombus burden patients (WMD = 0.15 × 10^9^/L; 95% CI = 0.08–0.23; *P* < .0001, *I*^2^ = 89%). On subgroup analyses, the pooled analyses showed a significant relationship between high monocyte count and high thrombus burden (WMD = 0.14 × 10^9^/L; 95% CI = 0.11–0.17; *P* < .00001) which shows that monocyte count is raised by 0.14 × 10^9^/L in the experimental group when compared with the control group. Studies having number of participants >300 (WMD = 0.25 × 10^9^/L; 95% CI = 0.17–0.32; *P* < .00001), participants <300 (WMD = 0.08 × 10^9^/L; 95% CI = 0.07–0.10; *P* < .00001), males >80% (WMD = 0.15 × 10^9^/L; 95% CI = 0.01–0.29; *P* = .03), males <80% (WMD = 0.15 × 10^9^/L; 95% CI = 0.04–0.27; *P* = .007) and study design as cross-sectional (WMD = 0.15 × 10^9^/L; 95% CI = 0.01–0.28; *P* = .04), cohort (WMD = 0.16 × 10^9^/L; 95% CI = 0.04–0.28; *P* = .01) showed a significant increase in monocyte count among high thrombus burden patients. However, the subgrouping analysis didn’t lead to any significant changes in the heterogeneity (*I*^2^ = 87%, *P* < .00001).

## 4. Discussion

Our study, which included 1426 STEMI patients who had PCI treatment, found that elevated monocyte counts were associated with a more significant thrombus load. Consistent with the results of previous studies, this analysis shows an increased monocyte count in STEMI patients with a high thrombus burden. To the authors’ best knowledge, this is the first meta-analysis to come up with a robust conclusion regarding this association.

Absolute thrombotic occlusion of the coronary artery due to atheromatous plaque rupture is a major pathophysiological event in STEMI. The time interval between MI diagnosis and myocardial reperfusion is crucial.^[16]^ PCI is the preferable treatment option if performed promptly, ideally within 120 minutes of primary medical care.^[17]^

Nevertheless, the management of thrombus burden during PCI is challenging. STEMI patients with high thrombotic burden, revealed a significantly high TIMI score after PCI, owing to the risks of major artery occlusion. A large thrombus burden in STEMI is an independent marker of mortality and is associated with frequent occurrence of the no-reflow phenomenon and stent thrombosis.^[18]^ Inflammation and oxidative stress are major players in the pathology of atherosclerosis and monocytes play a significant role in generating a systemic inflammatory response.^[19]^ The monocyte count within the circulating blood is a marker of progressive atherosclerotic disease and coronary artery disease.^[20]^

Studies have reported a high monocyte count in complications of atherosclerotic plaque which acts as a concatenation of STEMI.^[21]^ Out of the 5 studies, only 1^[13]^ showed a statistically insignificant association between low monocyte count and high thrombus burden which favors the results of our analysis. Overall, pooled results from our study revealed a statistically significant correlation between raised monocyte counts in the high thrombus burden STEMI individuals compared to the low thrombus burden group.

## 5. Limitations

Our analysis has several limitations which conveniently explain the heterogeneity. Firstly, 3 out of 5 studies^[11,12,14]^ show a direct association of monocyte count with thrombus burden, while the remaining 2 studies^[13,15]^ report a correlation of the MHR with patients having thrombotic burden; 2 studies^[11,14]^ included a monocyte cutoff value, 2 studies^[13,15]^ involved an MHR cutoff value and 1 study^[12]^ did not report a cutoff value which describes the increased heterogeneity. Secondly, the analysis was carried out based on the patient data mentioned in the selected studies and the prior antithrombotic history of individual patients might have affected the thrombotic status during PCI and resulted in clinical heterogeneity. Thirdly, the analysis consisted of prospective and retrospective cohorts and cross-sectional studies, and therefore randomized control trials on the subject are advised.

## 6. Conclusion

In conclusion, admission monocyte count is an independent and inexpensive marker of high intracoronary thrombus burden in patients with acute STEMI who had PCI treatment. This can help readily in the diagnosis and elimination of the risk of STEMI.

## Author contributions

**Conceptualization:** Hassan ul Hussain, Kanwal Ashok Kumar, Muhammad Husban Burney, Muqaddus Asif, Syeda Tayyaba Rehan, Khabab Abbasher Hussien Mohamed Ahmed, Irfan Ullah.

**Data curation:** Hassan ul Hussain, Kanwal Ashok Kumar.

**Formal analysis:** Hassan ul Hussain, Kanwal Ashok Kumar, Muqaddus Asif.

**Funding acquisition:** Hassan ul Hussain, Marium Zahid, Muhammad Husban Burney.

**Investigation:** Hassan ul Hussain, Muhammad Husban Burney, Muqaddus Asif.

**Methodology:** Hassan ul Hussain, Kanwal Ashok Kumar, Muqaddus Asif, Syeda Tayyaba Rehan.

**Project administration:** Hassan ul Hussain, Muhammad Husban Burney, Muqaddus Asif.

**Resources:** Hassan ul Hussain, Kanwal Ashok Kumar.

**Software:** Hassan ul Hussain, Marium Zahid.

**Supervision:** Hassan ul Hussain, Kanwal Ashok Kumar, Marium Zahid, Irfan Ullah.

**Validation:** Hassan ul Hussain, Kanwal Ashok Kumar, Marium Zahid, Muhammad Husban Burney, Muqaddus Asif, Syeda Tayyaba Rehan, Khabab Abbasher Hussien Mohamed Ahmed, Irfan Ullah.

**Visualization:** Hassan ul Hussain, Kanwal Ashok Kumar, Marium Zahid, Muhammad Husban Burney, Muqaddus Asif, Syeda Tayyaba Rehan, Khabab Abbasher Hussien Mohamed Ahmed, Irfan Ullah.

**Writing – original draft:** Hassan ul Hussain, Kanwal Ashok Kumar, Marium Zahid, Muhammad Husban Burney, Muqaddus Asif, Syeda Tayyaba Rehan, Khabab Abbasher Hussien Mohamed Ahmed, Irfan Ullah.

**Writing – review & editing:** Hassan ul Hussain, Kanwal Ashok Kumar, Marium Zahid, Muhammad Husban Burney, Muqaddus Asif, Syeda Tayyaba Rehan, Khabab Abbasher Hussien Mohamed Ahmed, Irfan Ullah.

## References

[1] Roth GA Johnson C Abajobir A. Global, regional, and national burden of cardiovascular diseases for 10 causes, 1990 to 2015. J Am Coll Cardiol. 2017;70:1–25. 28527533 10.1016/j.jacc.2017.04.052 PMC5491406

[2] Akbar H Foth C Kahloon RA Mountfort S. Acute ST elevation myocardial infarction. In: StatPearls. Treasure Island, FL: StatPearls Publishing; 2021. 30335314

[3] Toutouzas K Kaitozis O Tousoulis D. Primary percutaneous coronary intervention. Coronary Artery Dis Biol Clin Pract. 2017:417–37.

[4] Taleb S. Inflammation in atherosclerosis. Arch Cardiovasc Dis. 2016;109:708–15. 27595467 10.1016/j.acvd.2016.04.002

[5] Assessment of the Safety and Efficacy of a New Treatment Strategy with Percutaneous Coronary Intervention (ASSENT-4 PCI) investigators. Primary versus tenecteplase-facilitated percutaneous coronary intervention in patients with ST-segment elevation acute myocardial infarction (ASSENT-4 PCI): randomised trial. Lancet. 2006;367:569–78. 16488800 10.1016/S0140-6736(06)68147-6

[6] Rao SS Agasthi P. Thrombolysis in myocardial infarction risk score. In: StatPearls. Treasure Island, FL: StatPearls Publishing; 2022. 32310529

[7] Dutta P Nahrendorf M. Monocytes in myocardial infarction. Arterioscler Thromb Vasc Biol. 2015;35:1066–70. 25792449 10.1161/ATVBAHA.114.304652 PMC4409536

[8] Hansson GK. Inflammatory mechanisms in atherosclerosis. J Thromb Haemost. 2009;7(Suppl 1):328–31. 19630827 10.1111/j.1538-7836.2009.03416.x

[9] Hutton B Salanti G Caldwell DM. The PRISMA extension statement for reporting of systematic reviews incorporating network meta-analyses of health care interventions: checklist and explanations. Ann Intern Med. 2015;162:777–84. 26030634 10.7326/M14-2385

[10] Higgins JP Thompson SG. Quantifying heterogeneity in a meta-analysis. Stat Med. 2002;21:1539–58. 12111919 10.1002/sim.1186

[11] Separham A Abbaszadeh M Sakha H Sarvestani AH. Evaluation of the relation between monocyte count and angiographic thrombosis burden in patients with myocardial infarction with STEMI under PPCI treatment. J Res Clin Med. 2020;8:41.

[12] Zeeshan M Yousaf S Ahmed A. Co-relation of monocyte count in high vs. low thrombus burden ST-segment elevated myocardial infarction (STEMI) patients undergoing primary percutaneous coronary intervention. Cureus. 2022;14. 10.7759/cureus.24344 PMC9123895 35607551

[13] Arisoy A Altunkaş F Karaman K. Association of the monocyte to HDL cholesterol ratio with thrombus burden in patients with ST-segment elevation myocardial infarction. Clin Appl Thromb Hemost. 2017;23:992–7. 27534422 10.1177/1076029616663850

[14] Wang Z Liu N Ren L Lei L Ye H Peng J. Association of monocyte count on admission with the angiographic thrombus burden in patients with ST-segment elevation myocardial infarction undergoing primary percutaneous coronary intervention. Arq Bras Cardiol. 2018;110:333–8. 29538502 10.5935/abc.20180034 PMC5941955

[15] Sercelik A Besnili AF. Increased monocyte to high-density lipoprotein cholesterol ratio is associated with TIMI risk score in patients with ST-segment elevation myocardial infarction. Rev Port Cardiol. 2018;37:217–23. 29615294 10.1016/j.repc.2017.06.021

[16] Pantea-Roșan LR Pantea VA Bungau S. No-reflow after PPCI-A predictor of short-term outcomes in STEMI patients. J Clin Med. 2020;9:2956. 32932736 10.3390/jcm9092956 PMC7563881

[17] Stengaard C Sørensen JT Rasmussen MB Bøtker MT Pedersen CK Terkelsen CJ. Prehospital diagnosis of patients with acute myocardial infarction. Diagnosis (Berl). 2016;3:155–66. 29536903 10.1515/dx-2016-0021

[18] Sianos G Papafaklis MI Daemen J. Angiographic stent thrombosis after routine use of drug-eluting stents in ST-segment elevation myocardial infarction: the importance of thrombus burden. J Am Coll Cardiol. 2007;50:573–83. 17692740 10.1016/j.jacc.2007.04.059

[19] Cimmino G Loffredo FS Morello A. Immune-inflammatory activation in acute coronary syndromes: a look into the heart of unstable coronary plaque. Curr Cardiol Rev. 2017;13:110–7. 27758696 10.2174/1573403X12666161014093812 PMC5452145

[20] Afiune Neto A Mansur Ade P Avakian SD Gomes EP Ramires JA. Monocitose é um marcador de risco independente para a doença arterial coronariana [Monocytosis is an independent risk marker for coronary artery disease] [in Portuguese]. Arq Bras Cardiol. 2006;86:240–4. 16612453 10.1590/s0066-782x2006000300013

[21] Nozawa N Hibi K Endo M. Association between circulating monocytes and coronary plaque progression in patients with acute myocardial infarction. Circ J. 2010;74:1384–91. 20467155 10.1253/circj.cj-09-0779

